# HAX1 maintains the glioma progression in hypoxia through promoting mitochondrial fission

**DOI:** 10.1111/jcmm.17038

**Published:** 2021-11-10

**Authors:** Jinghui Lin, Yang Wang, Zhiqing Lin

**Affiliations:** ^1^ Department of Neurosurgery Ningbo First Hospital Ningbo China

**Keywords:** glioma, HCLS1‐associated protein X‐1, hypoxia, mitochondrial fission

## Abstract

HCLS1‐associated protein X‐1 (HAX1), an anti‐apoptotic molecular, overexpresses in glioma. However, the role of HAX1 in glioma cell surviving in hypoxic environment remains unclear. Western blotting, qRT‐PCR, Transwell assay, TUNEL assay, wounding healing assay, clone formation, tumour xenograft model and immunohistochemical staining were used to investigate the role of HAX1 in glioma. HAX1 regulated by HIF‐1α was increased in glioma cells cultured in hypoxia. Silencing of HAX1 could cause an increased apoptosis of glioma cells cultured in hypoxia. Silencing of HAX1 also decreased the proliferation, migration and invasion of glioma cells cultured in hypoxia. Increased mitochondrial fission could prevent glioma cells from the damage induced by HAX1 knockdown in hypoxia. Furthermore, HAX1 was found to regulate glioma cells through phosphorylated AKT/Drp signal pathway. In conclusion, our study suggested that HAX1 promoted survival of glioma cells in hypoxic environment via AKT/Drp signal pathway. Our study also provided a potential therapeutic target for glioma.

## 1 INTRODUCTION

Gliomas are known as the most common malignant tumours of the central nervous system (CNS) and express aggressive and malignant progression and high rates of recurrence.^1^, ^2^ Even though the traditional treatment methods (including surgical resection, radiotherapy and chemotherapy) have made considerable progress, the survival rate of glioma remains at a low level.^3^, ^4^, ^5^ A better understanding of glioma that provides a new potential treatment to glioma needs a breakthrough.

Tumour cells have multiple mechanisms for evading cell death through radiation therapy. These mechanisms include the ability of cancer cells to repair DNA damage caused by radiation and changes in the tumour microenvironment, such as hypoxia, elevated cytokine levels and transition of epithelial cells to mesenchymal cells.^6^ Hypoxia is a state of depriving tissues of enough oxygen supply and a common microenvironmental feature of almost all solid tumours.^7^ Hypoxia is also a pathophysiological status, usually due to the rapid proliferation of cancer cells beyond their blood supply, thus depleting nutrients and available oxygen.^8^ The hypoxic microenvironment has caused a great obstacle to the clinical effect of radiotherapy, because hypoxic tumours need three times the normal radiotherapy dose to achieve the cell death effect required by normal oxygen tumours.^9^ Glioma cell can adapt to hypoxic environment through various mechanisms,^10^, ^11^, ^12^ and hypoxia enhances glioma cell radiotherapy resistance, drug resistance and invasion ability. Hypoxia‐inducible factor 1α (HIF‐1α) facilitates the survival, invasion and proliferation of glioma by the release of DLK1 intracellular fragments and nuclear translocation.^13^ HIF‐1α also improves epithelia mesenchymal transition and chemoresistance of glioma through inducing ferritin light chain (FTL).^14^ Many molecules also regulate the characteristics of glioma in a hypoxic environment by HIF‐1α. For example, lncRNA H19 enhances glioma angiogenesis through HIF‐1α.^15^ Exosomes containing linc01060 can improve the progression of glioma by regulating MZF1/c‐Myc/HIF‐1α signalling pathway.^16^ ELTD1 promotes glioma proliferation, migration and invasion by stimulating JAK/STAT3/HIF‐1α signalling pathway.^17^ However, the mechanism of glioma in a hypoxic environment is still not fully understood.

HS‐1‐associated protein X‐1 (HAX1) was firstly reported as a protein to interact with HS‐1 20 years ago.^18^ HAX‐1 is a 35 kDa protein related to the cytoplasmic surface of mitochondria and endoplasmic reticulum (ER) membranes, but lacks appropriate transmembrane domains.^19^ Like HS1, HAX‐1 is a multifunctional protein that interacts with cytoplasmic surface proteins in the mitochondria. Through these interactions, HAX‐1 is thought to regulate mitochondrial membrane potential, seems to regulate calcium signalling.^20^ HAX1 regulates multiple pathophysiological processes and cellular metabolism, such as apoptosis, mitochondrial function, calcium homeostasis and cell migration.^20^, ^21^ The preliminary characterization of HAX1 reports the existence of the Bcl2 homology (BH) module, which is a protein motif found in the Bcl2 family of apoptosis regulatory proteins. HAX1 can promote the survival and metastasis of NSCLC by AKT/mTOR and MDM/P53 signalling pathway.^22^ Decreased expression of KDM4B induces mitochondrial apoptosis by reducing HAX1 expression in colorectal cancer.^23^ HAX1 also represses apoptosis in prostate cancer through inhibiting caspase‐9 activation.^24^ Importantly, recent studies reported that HAX1 was overexpressed in glioma and inhibits apoptosis of glioma.^25^, ^26^, ^27^ However, the role of HAX1 in glioma cell cultured in hypoxia remains unclear.

Mitochondria are also highly dynamic organelles that adapt to stress through various means. For example, mitochondria have a proteolytic system to degrade their misfolded proteins. Damaged mitochondrial outer membrane proteins can be degraded by the proteasome or eliminated by mitochondrial autophagy. Mitochondria maintain structural and functional integrity through continuous fusion and fission.^28^, ^29^ Therefore, mitochondrial fission is very important for the integrity of mitochondria. The promotion of mitochondrial fission can enhance tumour malignancy of cancer cells.^30^, ^31^, ^32^, ^33^, ^34^ Induced mitochondrial fission improves human lung cancer stem cell stemness by mitophagy.^33^ Meanwhile, promotion of mitochondrial fission is essential for Liver Cancer‐Initiating cell maintenance.^31^ Promotion of XBP1‐MARCH5‐MFN2 axis can inhibit mitochondrial fission and mitophagy to be against Melanoma cells.^34^ Drp‐1, a regulatory molecule of mitochondrial fission, was silenced to reduce the biological malignancy of glioma stem cells.^30^ What is more, inhibition of mitochondrial fission can enhance the effect of interference with NDUFA4L2 on glioma cells.^35^

In this study, we found that HAX1 was upregulated in glioma cell cultured in hypoxia. HIF‐1α could increase the expression of HAX1 to protect glioma cell in hypoxia. Silencing of HAX1 was also able to induce mitochondrial fission due to the damage of mitochondrial function, while induction of mitochondrial fission could restrain the effect of HAX1 knockdown on glioma cell cultured in hypoxia.

## 2 METHODS AND MATERIALS

### 2.1 Cell culture

Glioma cell lines T98G, U87 and U251 were obtained from Cell Bank of the Chinese Academy of Sciences (Shanghai, China). All experimental protocols were approved by the Human Ethics Committee of Ningbo First Hospital (Ningbo, China). U87 were cultured in MEM medium (Gibco) containing 1%NEAA (Gibco), 10% FBS (Gibco), 100 U/ml penicillin and 100 μg/ml streptomycin (Gibco). T98G and U251 were cultured in DMEM high‐glucose medium (Gibco) containing 1%NEAA (Gibco), 10% FBS (Gibco), 100 U/ml penicillin and 100 μg/ml streptomycin (Gibco). Cells were cultured at 37°C in a humidified atmosphere of 5% CO_2_, 95% air and 100% humidity or in hypoxic environments (1% O_2_).

### 2.2 RNA interference and vectors

Three small interference RNA to HAX1, HIF‐1α and HIF‐2α was established by GenePharma, China. The Lentivirus vector containing short hairpin RNAs (shRNA) knock‐down HAX1 (sh‐HAX1), Lentivirus knock‐down Negative Control (sh‐NC), lentivirus overexpress HAX1, Lentivirus overexpress Negative Control were designed and synthesized from Genome ditech (Shanghai, China). The sequences of HAX1, HIF‐1α and HIF‐2α were as follows: si‐HAX1‐1: Sense: 5’‐GGAUACGUUUCCACGAUAAdTdT‐3’, Antisense: 5’‐UUAUCGUGGAAACGUAUCCdTdT‐3’; si‐HAX1‐2: Sense: 5’‐GAGUGAUGCAAGAAGUGAAUU‐3’, Antisense: 5’‐UUCACUUCUUGCAUCACUCUU‐3’; si‐HAX1‐3: Sense: 5’‐UAAGGAGUCCGCAUGAUCGdTdT‐3’, Antisense: 5’‐CAGGUAGAGCACUGAAGCGGdTdT‐3’; Si‐HIF‐1α: Sense: 5’‐CUGAUGACCAGCAACUUGAdTdT‐3’, Antisense: 5’‐ UCAAGUUGCUGGUCAUCAGdTdT‐3’. Si‐HIF‐2α: Sense: 5’‐CACCGCCGTACTGTCAACCTCAAGTTTCAAGAGAACTTGAGGTTGACAGTACGGCTTTTTTG‐3’, Antisense: 5’‐GATCCAAAAAAGCCGTACTGTCAACCTCAAGTTCTCTTGAAACTTGAGGTTGACAGTACGGC‐3’. The doses HAX1 and HIF‐1α siRNA used were 50 nM according to manufacturer's protocol. The virus titre of lenti‐HAX1 and control virus are 1 × 10^8^ TU/ml. The medium was 2 ml, and the final concentration of virus was 1 × 10^6^ TU/ml.

### 2.3 RNA extraction and qRT‐PCR

Cells were lysed and total RNA was extracted using Trizol reagent (Beyotime, China) in accordance with manufacture's protocol. The cDNA was synthesized by reverse transcription kit (TaKaRa) according to manufacturer's protocol. QRT‐PCR was carried out with one‐step RT qPCR SYBRGreen Kit (DBI). The primers were purchased form Sangon Biotech and the sequences used were listed as follow^36^, ^37^: HAX1‐Forward: ACGCCTCGCTCAATTTCTCA, HAX1‐Reverse: AAGCCAAATTCCTCAGGGGG; HIF‐1α: HIF‐1α‐Forward: 5ʹ‐AGTGTACCCTAACTAGCCG‐3ʹ, HIF‐1α‐Reverse: 5ʹ‐CACAAATCAGCACCAAGC‐3ʹ. 2^−ΔΔCT^ methods were applied to calculate the mRNA expression levels.

### 2.4 Western blotting

Electrophoresis was performed by SDS‐PAGE gel and electro‐transfer was performed by a trans‐blot transfer slot (Bio‐Rad). PVDF membrane (Bio‐Rad) was blocked by 5% skimmed milk for 2 h at room temperature. The primary antibodies were added to membrane in accordance with protocols and incubated at 4℃ for 12 h. The primary antibodies include β‐actin (Beyotime, 1:1000), HAX1 (Proteintech, 1:1000), HIF‐1α (Proteintech, 1:1000), Drp1 (Proteintech, 1:1000), AKT (CST, 1:1000), p‐AKT (CST, 1:100) and p‐Drp1 (CST, 1:1000). Membranes were washed three times with TBST, and secondary antibodies (Proteintech, 1:1000) were added and incubated at room temperature for 1 h. ECL reagent (Millipore) was used to perform chemiluminescence detection.

### 2.5 TUNEL assay

Cells were seeded on the coverslips. 4% paraformaldehyde was used to fix cells and 0.3% Triton X‐100 was used to penetrate cells for 5 min. 50 μL TUNEL reagent was added to coverslip and incubated for 1 h. Images were taken by microscopy (Olympus BX51).

### 2.6 Transwell assay

Transwell chamber and Matrigel were gained form Corning (USA). The lower chamber was added with 500 μl culture medium containing 10% serum, and serum‐free medium containing 5 × 10^4^ cells was added into the upper chamber and cultured for 24 h in hypoxic environments (1% O_2_). The invasion rate was counted after the invaded cells were stained with 0.1% crystal violet (Sinopharm Chemical Reagent).

### 2.7 Wounding healing assay

Cells were seeded into 6 cm plate overnight in a consistent and humidified atmosphere of 5% CO_2_ at 37℃. A vertical scratching was made by the tip of the pipette and cells were incubated in medium containing 1% serum for 24 h. The relative migration distance of cells was evaluated by a Leica DMI3000 B inverted microscope.

### 2.8 Clone formation

Colone formation was carried out following previously published methods.^38^ Briefly, cells during logarithmic phases were added into in six‐well plates at a density of 1000 cells per well. Two weeks later, we fixed cells by methanol for 30 min. Colonies were then stained by using 0.1% crystal violet (Beyotime), and the relative numbers of colonies were manually calculated.

### 2.9 Tumour xenograft model

Five male BALB/C nude mice (18–20 g) purchased from Shanghai SLAC Laboratory Animal Co., Ltd. were subcutaneously injected with U87 cells transfected with Lenti‐shNC or Lenti‐shHAX1. After 1 month, mice were sacrificed and tumours were excised. Tumours were weighed and used to perform immunohistochemical assays. The volume of tumour was measured per 5 days. This information was added in the revised manuscript. Tumour sizes were calculated with the formula: (mm^3^) = (L × W2) × 0.5.

### 2.10 Immunohistochemical staining

The tumour specimens were fixed with 4% formaldehyde, embedded in paraffin, and cut into 4 μm sections for immunohistochemical analysis. Briefly, 3% hydrogen peroxide was used to block the endogenous peroxidase activity for 10 min, and then, 5% BSA was used to block the non‐specific binding sites for 30 min at room temperature. Primary antibodies were incubated overnight at 4℃. For primary antibodies, we used PCNA (1:100, Proteintech), HAX1 (1:150, Proteintech), p‐Drp (1:200, Proteintech) and p‐AKT (1:200, CST). Sections were incubated with an appropriate HRP‐conjugated secondary antibody (Santa Cruz Biotechnology) and counterstained with haematoxylin.

### 2.11 Mitochondrial morphology

When the cells were cultured to a certain density in a cell culture plate, remove the cell culture solution, add the Mito‐Tracker Red CMXRos (Beyotime) working solution, and incubate at 37°C for 15–30 min. Remove the Mito‐Tracker Red CMXRos working solution and add fresh cell culture solution pre‐incubated at 37°C. Observe with a fluorescence microscope. At this time, mitochondrial morphology can be observed as bright and intense fluorescent staining.

### 2.12 Statistical analysis

SPSS 20.0 statistical software was used to perform statistical analysis. The measurement data are expressed as x ± s. The independent sample *t* test was used for comparison between the two groups. One‐way analysis of variance (ANOVA) was used for comparison between groups, followed by Tukey multiple comparisons. *p* < 0.05 is of systematic significance. *p *< 0.05. *p < 0.05, **p < 0.01, ***p < 0.001.

## 3 RESULTS

### 3.1 Hypoxia induces HAX1 expression in gliomas

**FIGURE 1:**
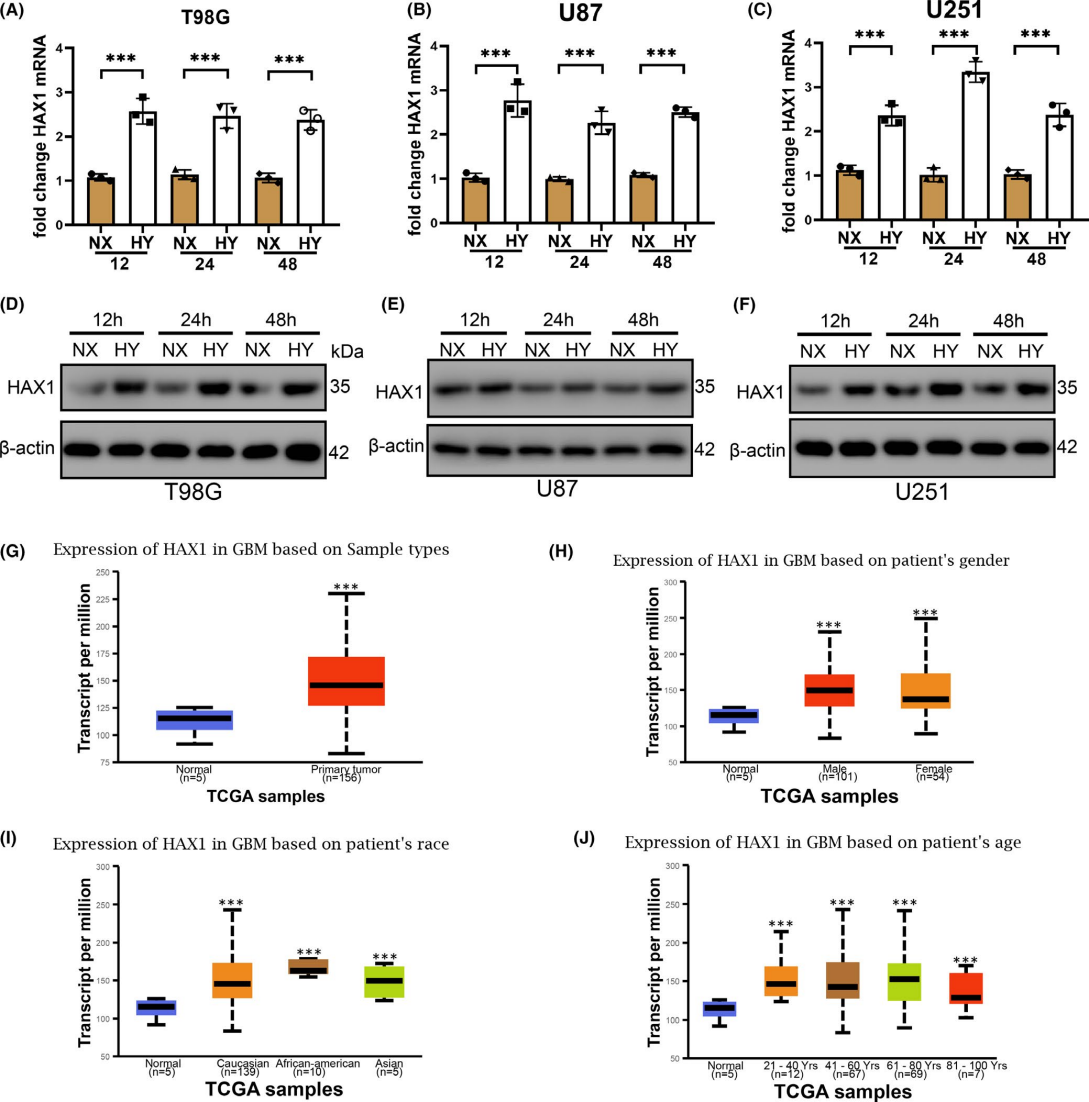
High HAX1 expression was observed in glioma cells cultured in hypoxia. T98G, U87 and U251 cells were cultured in normoxia and hypoxia for 12, 24 and 48 h. (A) HAX1 mRNA expression was detected by qRT‐PCR in T98G cells. (B) HAX1 mRNA expression was detected by qRT‐PCR in U87 cells. (C) HAX1 mRNA expression was detected by qRT‐PCR in U251 cells. (D) HAX1 protein expression was detected by Western blotting in T98G cells. (E) HAX1 protein expression was detected by Western blotting in U87 cells. (F) HAX1 protein expression was detected by Western blotting in U251 cells. (G) Expression of HAX1 in GBM based on sample. (H) Expression of HAX1 based on patient's gender. (I) Expression of HAX1 in GBM based on patient's race. (J) Expression of HAX1 in GBM based on patient's age

To investigate whether hypoxia induces HAX1 expression, glioma cell line T98G, U87 and U251 were cultured in hypoxia for 12, 24 and 48 h. HAX1 mRNA and protein expression were increased remarkably in hypoxia (Figure 1A–E and F). However, the expression of HAX1 is not time dependent in hypoxia. Therefore, follow‐up experiments were performed in glioma cells cultured in hypoxia for 24 h. To further confirm the expression of HAX1 in human gliomas. We clarified the expression of HAX1 in human gliomas by TCGA analysis database: http://ualcan.path.uab.edu/index.html. As was shown in Figure 1G–J, HAX1 mRNA expression was higher in gliomas that normal tissue. Gender has no difference in the effect of HAX1 expression in glioma. No difference in race and age. Deng et al showed the same results that HAX1 expression was increased in glioma tissues.^25^ These results showed that HAX1 expression was overexpression in gliomas and hypoxia could induce HAX1 expression in glioma cell lines.

### 3.2 HAX1 knockdown inhibits the proliferation, invasion and migration of glioma cell as well as cause apoptosis of glioma cells

**FIGURE 2:**
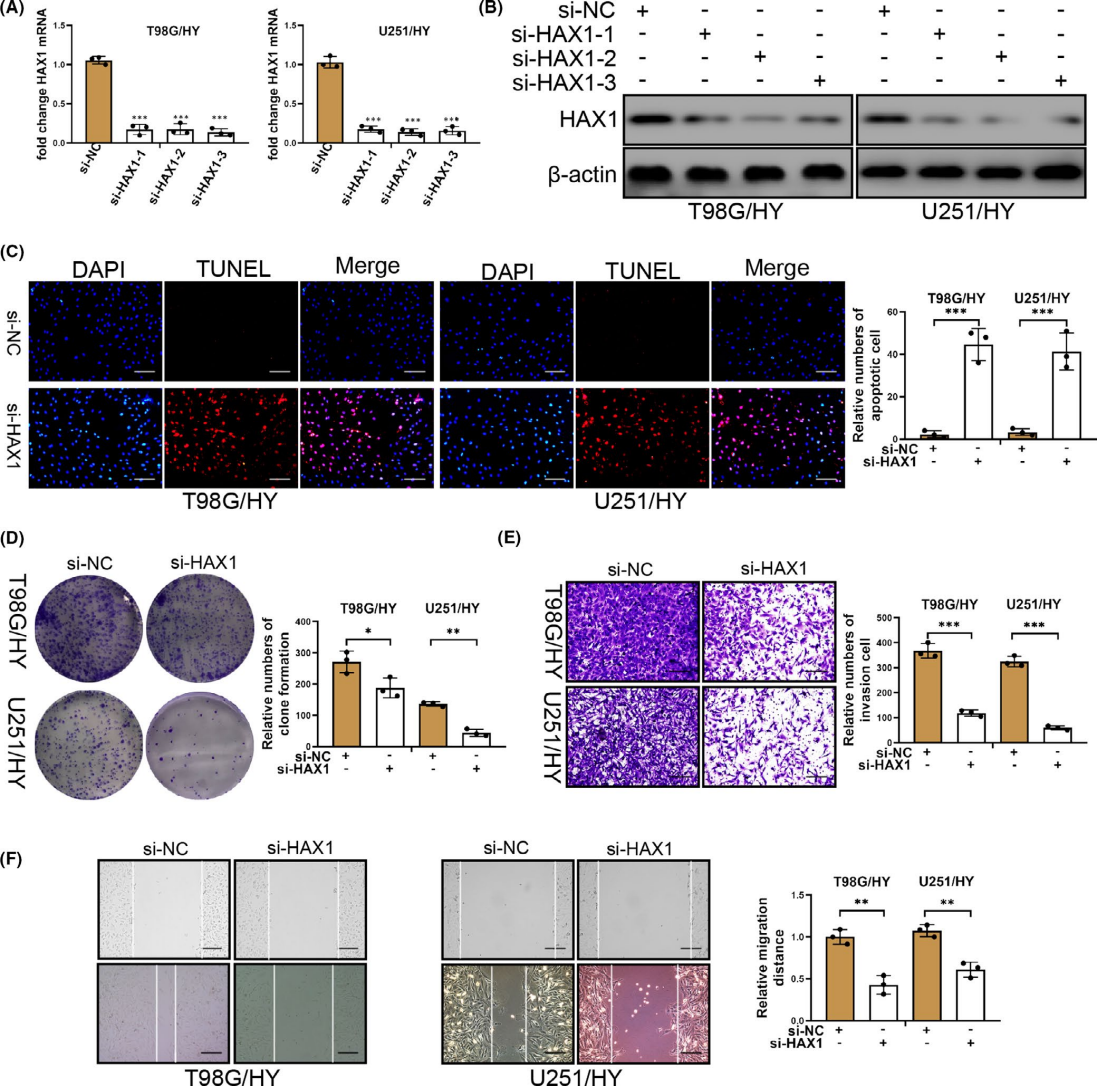
Silencing of HAX1 reduced the adaption of glioma cells to hypoxia. HAX1 was knockdown in glioma cells cultured in hypoxia. (A) The effect of small interference RNA to HAX1 was confirmed by qRT‐PCR and HAX1 mRNA expression was reduced in T98G and U251 cells in hypoxia. (B) The effect of small interference RNA to HAX1 was confirmed by Western blotting and HAX1 protein expression was reduced in T98G and U251 cells in hypoxia. (C) T98G and U251 cell apoptosis transfected with si‐NC or si‐HAX1 were determined by TUNEL staining. Scale bar = 50 μm. (D) The proliferation of T98G and U251 cells transfected with si‐NC or si‐HAX1 was measured by colony formation. (E) T98G and U251 cell invasion was detected by Transwell assay after si‐NC or si‐HAX1 transfection. Scale bar = 50 μm. (F) T98G and U251 cell migration was determined by wounding healing after si‐NC or si‐HAX1 transfection. Scale bar = 100 μm. T98G/HY: T98G cells were cultured in hypoxia. U251/HY: U251 cells were cultured in hypoxia

To further determine the role of HAX1 in glioma cell cultured in hypoxia, we designed small interference RNA (si‐RNA) to silence the expression of HAX1. The effect of si‐HAX1 on glioma cell was confirmed by qRT‐PCR and Western blotting. As was shown in Figure 2A,B, HAX1 mRNA expression was reduced by si‐HAX1‐1, si‐HAX‐2 and si‐HAX‐3 in T98G and U251 cells cultured in hypoxia (T98G/HY and U251 HY). HAX1 protein expression also was decreased by si‐HAX1‐1, si‐HAX‐2 and si‐HAX‐3 in T98G/HY and U251/HY cells. Si‐HAX1‐1 was used in follow‐up experiments. In subsequent experiment, silence of HAX1 was able to induce T98G/HY and U251/HY cell apoptosis (Figure 2C). As an anti‐apoptotic protein, silence of HAX1 caused T98G/HY and U251/HY cell apoptosis, which was consistent with early discovery.^22^, ^24^, ^39^, ^40^ In addition, silencing of HAX1 also caused a decreased invasion, proliferation and migration of T98G/HY and U251/HY cells (Figure 2D–F). These results demonstrated that HAX1 knockdown could reduce the proliferation, invasion and migration of glioma cells cultured in hypoxia. HAX1 knockdown also promoted the apoptosis of glioma cells in hypoxia.

### 3.3 HIF‐1α knockdown reduces HAX1 expression in glioma cell in hypoxia

**FIGURE 3:**
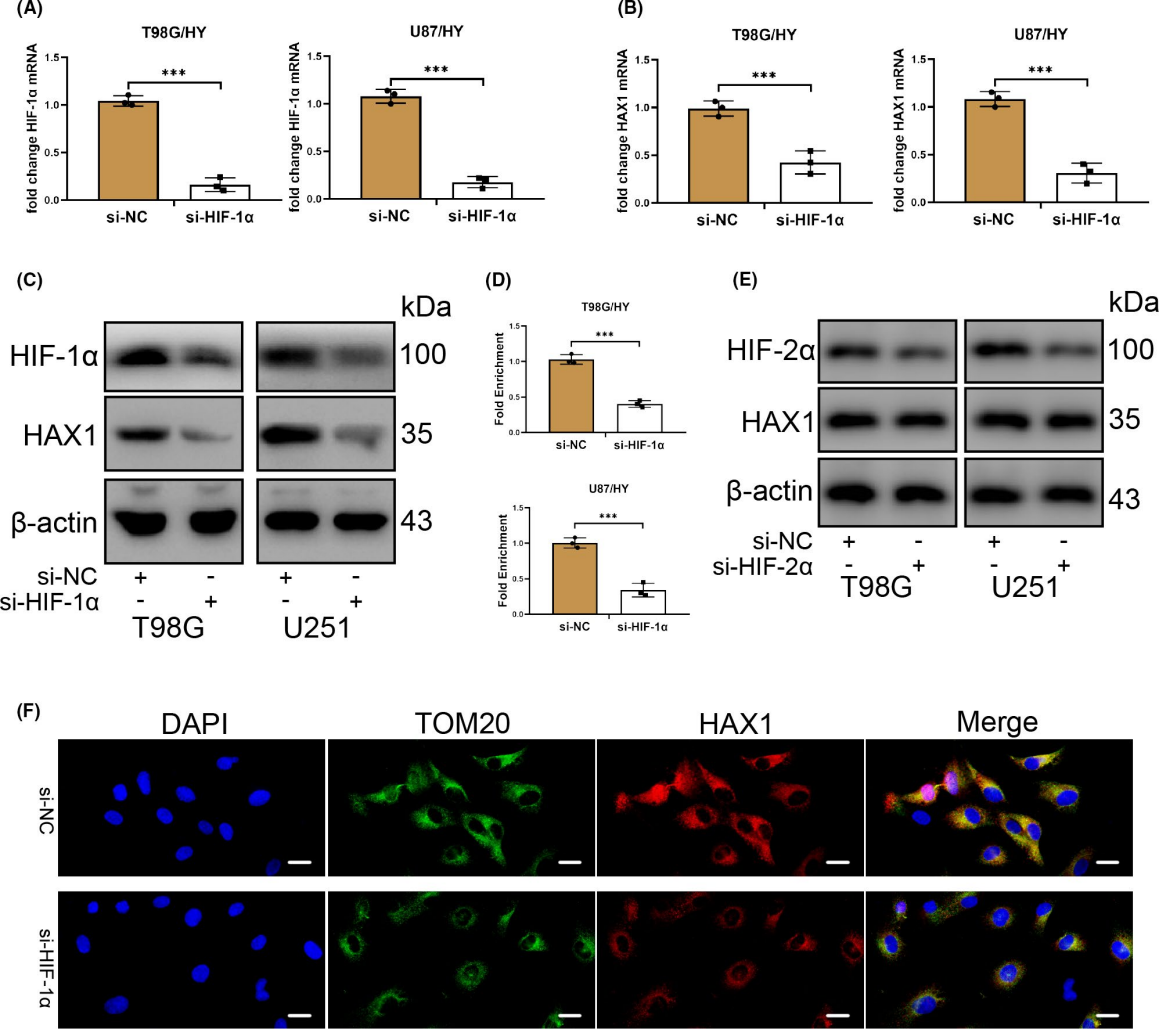
Inhibition of HIF‐1α resulted in the reduction of HAX1 in glioma cells cultured in hypoxia. HIF‐1α was knockdown in glioma cells cultured in hypoxia. (A) The effect of small interference RNA to HIF‐1α was confirmed by qRT‐PCR and HIF‐1α mRNA expression was reduced in T98G and U87 cells in hypoxia. (B) HAX1 mRNA expression in T98G and U87 cells was measured by qRT‐PCR after si‐NC or si‐HIF‐1α transfection. (C) The effect of small interference RNA to HIF‐1α was confirmed by Western blotting and HIF‐1α and HAX1 protein expression was reduced in T98G and U87 cells in hypoxia. (D) The results of CHIP were confirmed by qRT‐PCR. (E) The effect of small interference RNA to HIF‐2α was confirmed by Western blotting and HIF‐2α protein expression was reduced in T98G and U87 cells in hypoxia. There was no change of HAX1 protein expression. (F) TOM20 and HAX1 protein expression and location were determined by immunofluorescence staining after si‐NC or si‐HIF‐1α transfection. Scale bar = 10 μm. T98G/HY: T98G cells were cultured in hypoxia. U87/HY: U87 cells were cultured in hypoxia

HIF‐1α (hypoxia‐inducible factor 1 subunit α) can improve the survival ability and characteristics of various types of tumour cells or organs in a hypoxic environment.^38^, ^41^, ^42^, ^43^, ^44^ Therefore, we speculated that HAX1 served as downstream of HIF‐1α to regulate glioma cell in hypoxia. To confirm our hypothesis, si‐HIF‐1α was designed to silence HIF‐1α expression. The results of qRT‐PCR showed that HIF‐1α mRNA was reduced by si‐HIF‐1α (Figure 3A). HIF‐1α knockdown also caused a decreased mRNA expression of HAX1 (Figure 3B). Then, Western blotting was performed to detect the protein expression of HIF‐1α and HAX1. HIF‐1α protein expression was reduced significantly in T98G/HY and U87/HY cells transfected with si‐HIF‐1α (Figure 3C). The decreased HAX1 protein expression was also caused by si‐HIF‐1α (Figure 3C). CHIP experiments had been performed and the results showed that HIF‐1α upregulated the expression of HAX1 by interacting to the HAX1 promoter (Figure 3D). To confirm whether HIF‐2α was involved in regulating HAX1 expression, HIF‐2α was silenced by si‐HIF‐2α. The results showed that HIF‐2α did not affect the regulation of HAX1 (Figure 3E). Immunofluorescence results showed that HIF‐1α knockdown could inhibit the HAX1 protein expression (Figure 3F). These results indicated that HAX1 acted as a downstream of HIF‐1α to maintain the survival of glioma cell.

### 3.4 HAX1 acts as a downstream of HIF‐1α in glioma cell in hypoxia

**FIGURE 4:**
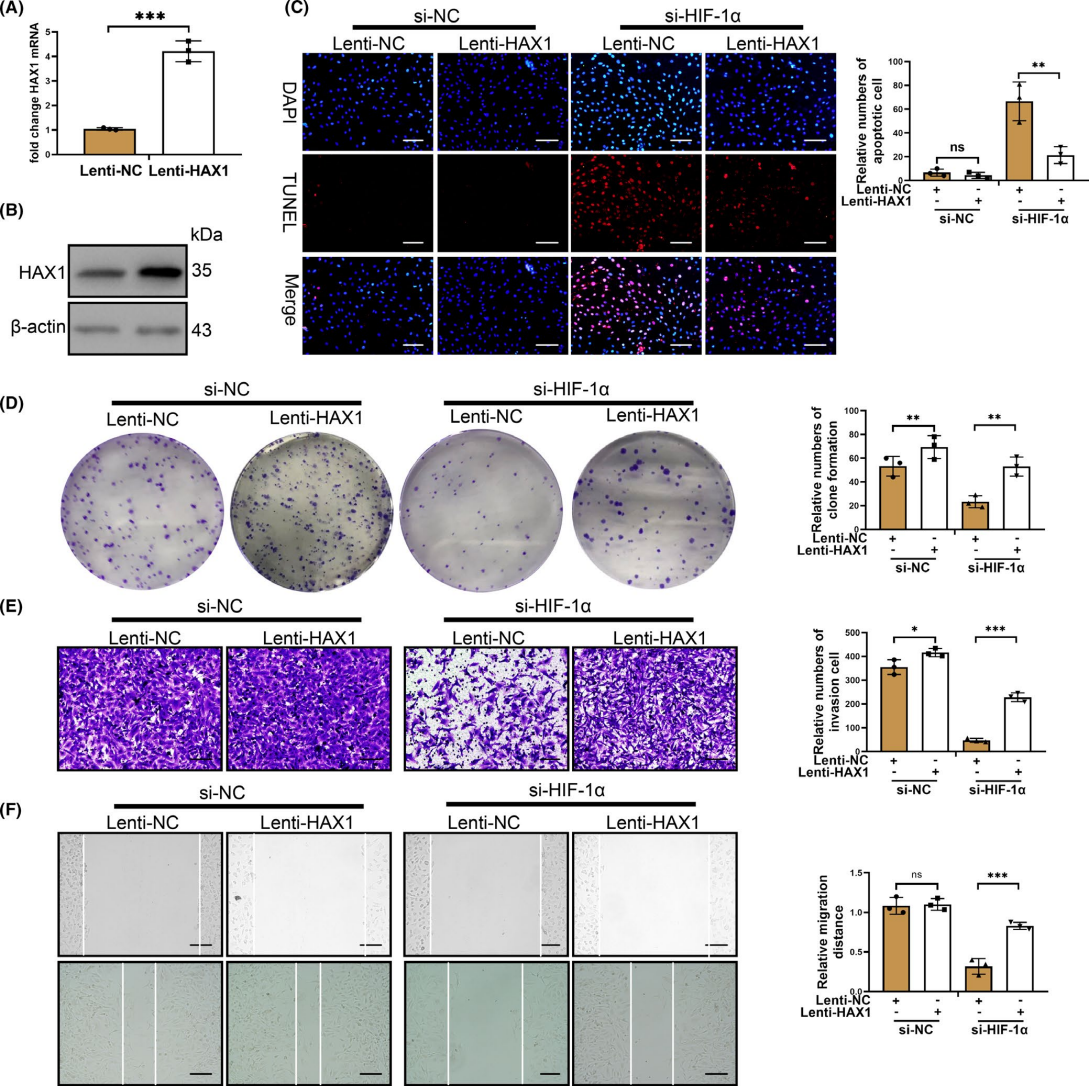
Upregulation of HAX1 protected glioma cells from HIF‐1α knockdown in hypoxia. T98G cells were transfected with si‐HIF‐1α after transfection of Lenti‐HAX1 in hypoxia. (A) The effect of Lentivirus overexpress HAX1 on T98G cells was confirmed by qRT‐PCR and HAX1 mRNA expression was upregulated in T98G cells after Lenti‐HAX1 transfection in hypoxia. (B) The effect of Lentivirus overexpress HAX1 on T98G cells was confirmed by Western blotting and HAX1 protein expression was upregulated in T98G cells after Lenti‐HAX1 transfection in hypoxia. (C) T98G apoptosis was determined by TUNEL staining. The experiment groups were shown in the figure. Scale bar = 50 μm. (D) The proliferation of T98G cells transfected was measured by colony formation. The experiment groups were shown in the figure. (E) T98G invasion was detected by Transwell assay. The experiment groups were shown in the figure. Scale bar = 50 μm. (F) T98G cell migration was determined by wounding healing. The experiment groups were shown in the figure. Scale bar = 100 μm.

To further investigate whether HAX1 serves as a downstream of HIF‐1α to maintain the survival ability and characteristics of glioma, we established LV3‐pGLV‐H1 + Puro plasmids with pcDNA‐HAX1 or control oligonucleotides (Lenti‐HAX1 and Lenti‐NC) to overexpress HAX1 protein expression. As was shown in Figure 4A and B, Lenti‐HAX1 was able to upregulate HAX1 expression in T98G/HY cells. HIF‐1α knockdown caused T98G/HY cell apoptosis while overexpression of HAX1 was capable of reducing T98G/HY cell apoptosis transfected with si‐HIF‐1α (Figure 4C). Subsequently, we found HIF‐1α knockdown also decreased the proliferation of T98G/HY cells by Clone formation assay (Figure 4D). HAX1 upregulation could improve mildly the clone formation of T98G/HY cells and promote significantly the clone formation of T98G/HY cells transfected with si‐HIF‐1α (Figure 4D). HAX1 overexpression could also slightly promote the invasion of T98G/HY cells and relieve si‐HIF‐1α‐induced inhibition of invasion (Figure 4E). For the migration of T98G/HY cells, HAX1 overexpression could only alleviate the effect of si‐HIF‐1α on T98G/HY (Figure 4F). These results demonstrated that HAX1 acted as a downstream of HIF‐1α in glioma cell in hypoxia.

### 3.5 HAX1 knockdown decreases mitochondrial fission of glioma cells cultured in hypoxia

**FIGURE 5:**
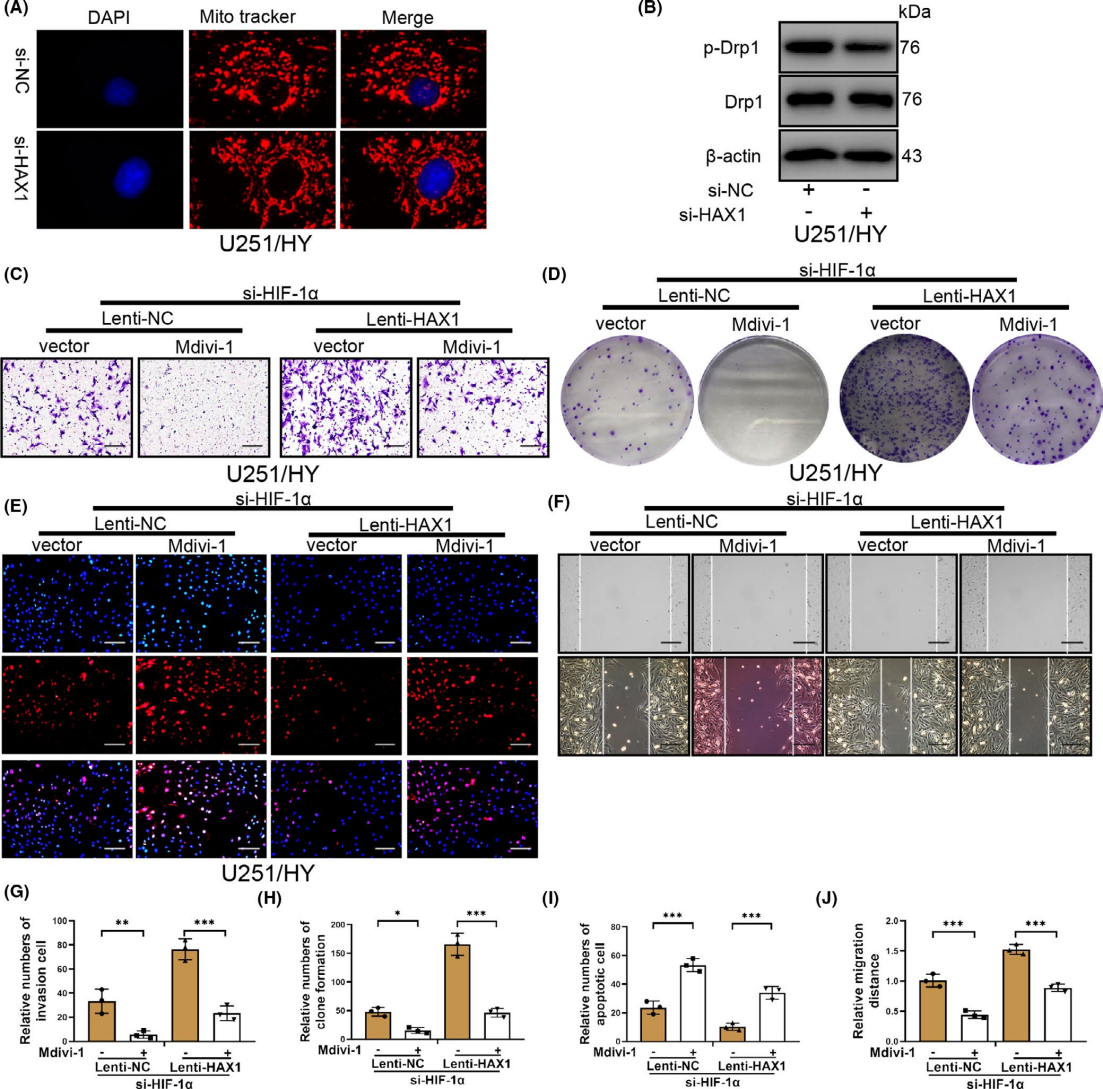
HAX1 protected glioma cells in hypoxia via mitochondrial fission. U251 cells were treated with mitochondrial fission inhibitor Mdivi‐1 after si‐HIF‐1α and Lenti‐HAX1 transfection. (A) Mitochondrial morphology in U251 cells was traced by Mito‐tracker after HAX1 was silenced. (B) The protein levels of p‐Drp, Drp and β‐actin were measured by Western blotting after HAX1 was silenced. (C) U251 cell invasion was detected by Transwell assay. The experiment groups were shown in the figure. Scale bar = 50 μm. (D) The proliferation of U251 cells transfected was measured by colony formation. The experiment groups were shown in the figure. (E) U251 apoptosis was determined by TUNEL staining. The experiment groups were shown in the figure. Scale bar = 50 μm. (F) U251 cell migration was determined by wounding healing. The experiment groups were shown in the figure. Scale bar = 100 μm. (G–J) Quantitative analysis to invasion, proliferation, apoptosis and migration of U251 cells. U251/HY: U251 cells were cultured in hypoxia

Mitochondrial fission is a double‐edged sword that can protect and damage cells.^45^, ^46^, ^47^, ^48^, ^49^ Previous studies have reported that mitochondrial fission can protect hepatocellular carcinoma cell from hypoxia^48^ and promote cisplatin resistance of ovarian cancer.^47^ However, mitochondrial fission can repress breast cancer stem cell activity and cause annulus fibrosus cell and nucleus pulposus apoptosis.^45^, ^46^ HAX1 is highly expressed in mitochondria, regulates mitochondrial metabolism and apoptosis induced by mitochondrial pathways.^21^, ^50^, ^51^ Mito tracker assays showed that silencing of HAX1 decreased significantly mitochondrial fission (Figure 5A). Silencing of HAX1 also decreased the protein level of p‐Drp1 in U251/HY cells (Figure 5B). To further confirm the role of mitochondrial fission, we used the mitochondrial fission inhibitor Midivi‐1 to treat U251/HY cells transfected with Lenti‐NC or Lenti‐HAX1. As was shown in Figure 5C,G, Midivi‐1 could reduce the invasion of U251/HY cells transfected with Lenti‐NC and the protective effect of Lenti‐HAX1 in the invasion of U251/HY cells. Inhibition of mitochondrial fission by Midivi‐1 was also able to decrease further the proliferation and migration of U251/HY cells (Figure 5D,F,H,J). The reduction of U251/HY cell apoptosis caused by si‐HIF‐1α also was inhibited by Midivi‐1 (Figure 5E,I). These results confirmed that mitochondrial fission induced by HAX1 could protect U251 cells cultured in hypoxia.

### 3.6 HAX1 can protect glioma cells through AKT/Drp signal pathway in hypoxia

**FIGURE 6:**
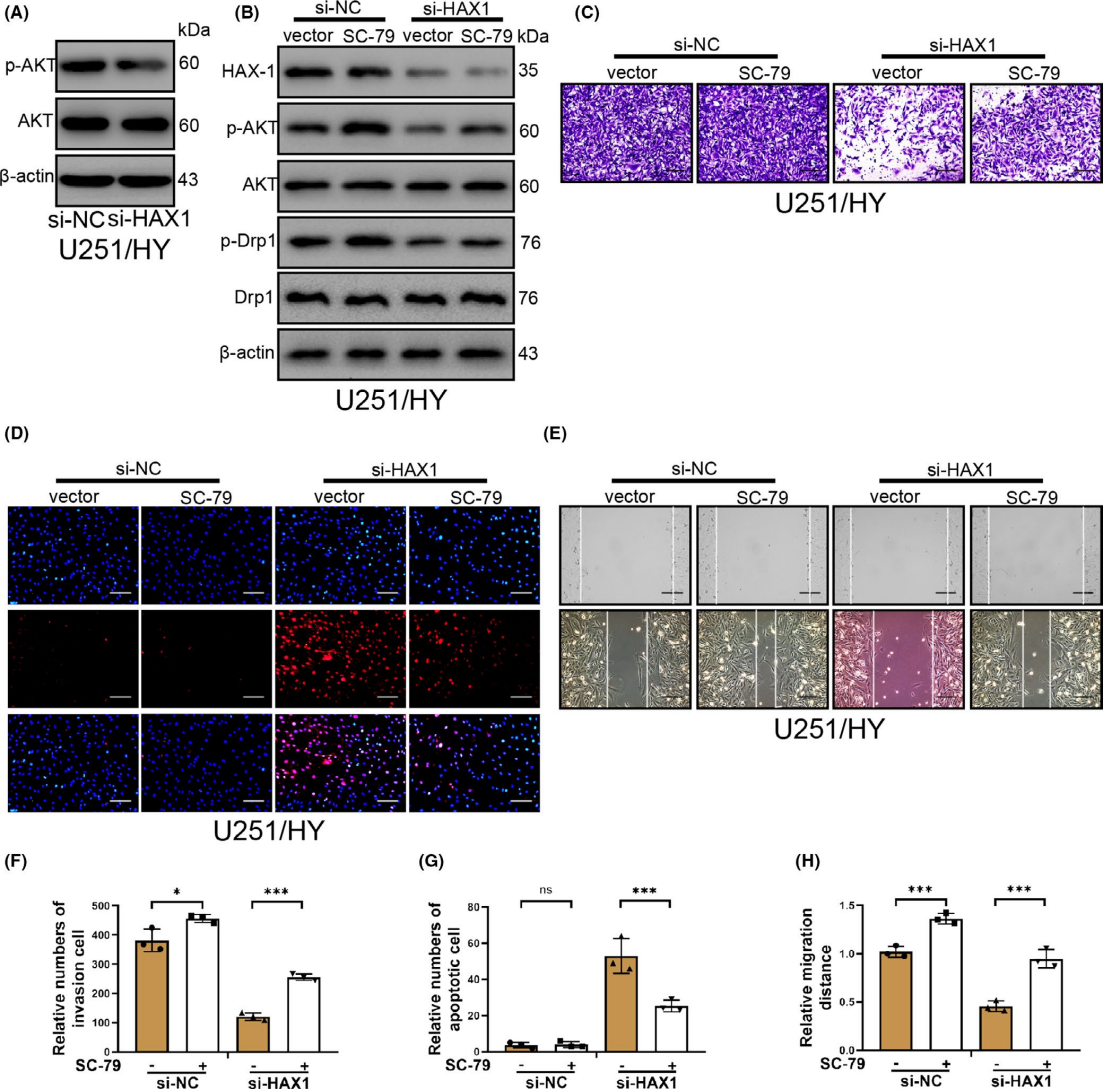
HAX1 regulated phosphorylated Drp through AKT signal pathway. U251 cells in hypoxia were treated with SC‐79 after si‐NC or si‐HAX1 transfection. (A) The protein levels of p‐AKT, AKT and β‐actin were measured by Western blotting after HAX1 was silenced. (B) The protein levels of HAX1, p‐Drp, Drp, p‐AKT, AKT and β‐actin were measured by Western blotting. The experiment groups were shown in the figure. (C) U251 cell invasion was detected by Transwell assay. The experiment groups were shown in the figure. Scale bar = 50 μm. (D) U251 apoptosis was determined by TUNEL staining. The experiment groups were shown in the figure. Scale bar = 50 μm. (E) U251 cell migration was determined by wounding healing. The experiment groups were shown in the figure. Scale bar = 100 μm. (F–H) Quantitative analysis to invasion, apoptosis and migration of U251 cells. U251/HY: U251 cells were cultured in hypoxia

HAX1 can promote the survival and metastasis of NSCLC by AKT/mTOR and MDM/P53 signalling pathway.^19^ To further investigate whether HAX1 enhances the mitochondrial fission by AKT/Drp signal pathway, we detected the protein level of p‐AKT after HAX1 knockdown and found that HAX1 knockdown reduced the level of p‐AKT in glioma cells (Figure 6A). And then, we used p‐AKT agonist SC‐79 (5 µg/ml for 1 h) to treat glioma cells after HAX1 knockdown. SC‐79 did not change HAX1 expression in U251/HY cells transfected with si‐NC or si‐HAX1 (Figure 6B). Secondly, Transwell assays, wounding healing assays and TUNEL assays were used to investigate the invasion, migration and apoptosis of U251/HY cells transfected with si‐NC or si‐HAX1. SC‐79 increased obviously the invasion of U251/HY cells transfected with si‐HAX1 (Figure 6C,F). SC‐79 increased the migration of U251/HY cells transfected with si‐HAX1 or si‐NC (Figure 6E,H). On the contrary, SC‐79 reduced significantly the apoptosis of U251/HY cells induced by si‐HAX1 (Figure 6D,G). These results indicated that HAX‐1 promoted the mitochondrial fission via AKT/Drp signal pathway on U251/HY cells.

### 3.7 HAX1 knockdown represses glioma growth in vivo

**FIGURE 7:**
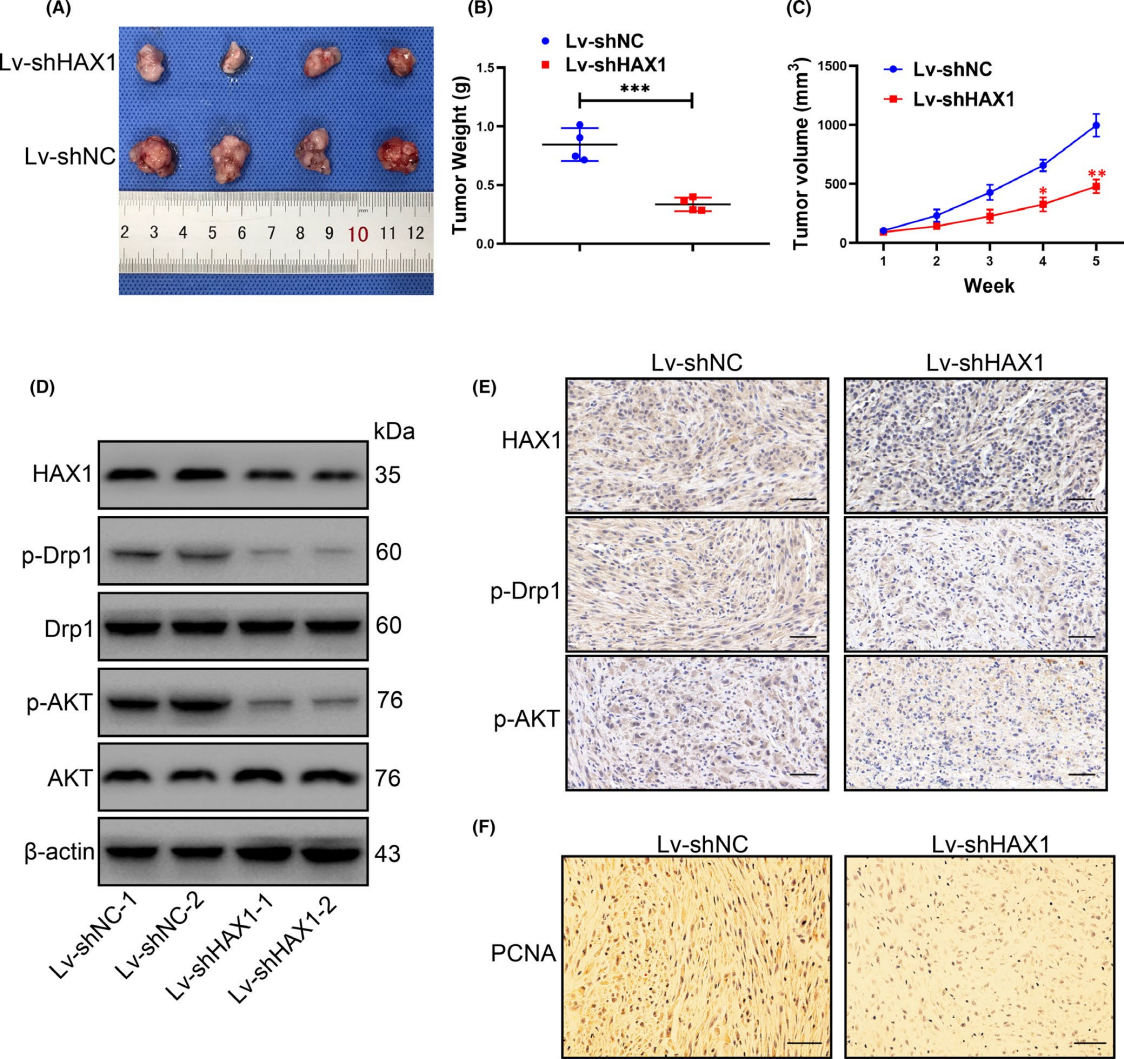
HAX1 knockdown reduced the growth of glioma *in vivo*. Glioma cells transfected with Lv‐shNC or Lv‐shHAX1 were grown in nude mice. (A) The tumour containing Lv‐shNC or Lv‐shHAX1 were shown. (B) The average weight of tumour in each group was recorded. (C) Tumour growth curves for each week were indicated. (D) The protein expression of HAX1, p‐Drp, Drp, p‐AKT, AKT and β‐actin in tumours was measured by Western blotting. (E) The protein expression of HAX1, p‐Drp and p‐AKT in tumours was measured by immunohistochemical staining. Scale bar = 20 μm. (F) The protein expression of PCNA in tumours were measured by immunohistochemical staining. Scale bar = 20 μm

HAX1 function in vivo was evaluated in BALB/c nude mice xenografted with U87 cells. U87/HY cells were transfected with Lenti‐shNC or Lenti‐shHAX1. The growth of glioma tumour was slower after HAX1 knockdown (Figure 1A). Tumour weight also was reduced by HAX1 knockdown (Figure 7B). HAX1 knockdown significantly reduced tumour volume of glioma tumour (Figure 7C). HAX1 protein expression was decreased by Lenti‐shHAX1 in U87/HY cells (Figure 7D). Moreover, the protein levels of p‐Drp and p‐AKT were also decreased in Lenti‐shHAX1 groups (Figure 7D). The results of immunohistochemical staining confirmed that HAX1, p‐Drp and p‐AKT protein levels were reduced in the tissues after HAX1 knockdown (Figure 7E). There was a decrease of PCNA expression in Lenti‐shHAX1 groups which indicated HAX1 knockdown inhibited the tumour growth (Figure 7F). These results revealed that HAX1 knockdown induced the inhibition of growth of glioma tumours.

## 4 DISCUSSION

Glioma is the most common intracranial malignant tumour, which poses a serious threat to human health.^52^ According to WHO classification, glioma can be divided into Ⅰ‐Ⅴ grades, Ⅰ and Ⅱ namely low‐grade glioma (LGG), Ⅲ‐Ⅳ high‐grade glioma (HGG). Among them, glioblastoma multiforme (GBM) has the highest malignant degree. The average survival time of Glioblastoma multiforme (GBM) is only about 1 year. Some studies have reported that its 5‐year survival rate is only 9.8%.^53^ At present, surgical resection, postoperative adjuvant radiotherapy, chemotherapy, immunotherapy, photodynamic therapy, electric field therapy and other effective treatment methods for glioma are still the first choice, but the prognosis of patients is still poor. Genome‐wide molecular maps have revealed the genetic and epigenetic biological characteristics of different types of gliomas, and their clinical value in the diagnosis, prognosis and treatment of gliomas has attracted widespread attention.^54^ Therefore, it is of great significance to find useful molecular markers for clinicians to optimize treatment and improve the therapeutic effect of glioma patients.^55^

HAX1, widely expressed in many human organs, was preferentially located in mitochondria, and a small part in the endoplasmic reticulum and nuclear membrane.^56^ The molecular weight of HAX1 is 35 kD, and the whole protein is composed of 279 amino acid residues. Our results demonstrated that higher expression of HAX1 was found in glioma cells survived in hypoxia. HAX1 knockdown could cause a decrease of proliferation, migration and invasion of glioma cells cultured in hypoxia. HAX1 also increased apoptosis of glioma cells cultured in hypoxia. HAX1 can promote the survival and metastasis of NSCLC by AKT/mTOR and MDM/P53 signalling pathway.^22^ HAX1 also represses apoptosis in prostate cancer through inhibiting caspase‐9 activation.^24^ Importantly, recent studies reported that HAX1 was overexpressed in glioma and inhibits apoptosis of glioma.^25^, ^26^, ^27^ These results indicated that HAX1 was able to maintain the survival of glioma cells in hypoxia.

As a key molecule in the regulation of hypoxia, HIF‐1α promotes cells to adapt to the hypoxic environment by changing the way of cell energy supply and mitochondrial metabolism.^41^, ^57^, ^58^ Therefore, we first tested whether HIF‐1α regulated the expression of HAX1 in glioma. HIF‐1α was discovered to improve the expression of HAX1 in glioma cells cultured in hypoxia. Overexpression of HAX1 could attenuate the phenotypic changes caused by HIF‐1α knockdown. These results demonstrated that HAX1 acted as a target of HIF‐1α to regulate glioma cells in hypoxia.

HAX1 was preferentially located in mitochondria, which showed that HAX1 knockdown might have an important effect on mitochondria. Therefore, we examined the morphology of mitochondria and found that silencing of HAX1 was capable of inhibiting mitochondrial fission. Decreased expression of phosphorylated Drp caused by HAX1 was found in glioma cells. Inhibition of mitochondrial fission could reduce the effect of HAX1 in glioma cells. The sequences of the N‐terminal amino acid residues were similar to those of the Bcl‐2 protein family, including two BH domains (BH1, AA40‐56; BH2, aa74–89).^59^ Previous studies have shown that the anti‐apoptotic effect of Bcl‐2 is regulated by its phosphorylation. When Bcl‐2 is phosphorylated at a partial site of a non‐structural ring within the molecule, its anti‐apoptotic activity decreases, while the loss of this non‐structural ring or the mutation of the phosphorylation site within it will lead to an increase in its anti‐apoptotic activity.^60^, ^61^, ^62^ Besides, mitochondrial quality control requires mitophagy and mitochondrial fission. Mitochondrial fission can induce mitophagy to inhibit cell apoptosis.^63^ Reduced dynamin‐related protein 1‐related mitophagy was able to induce myocardial apoptosis in the ageing heart. Silencing of NDUFA4L2 causes mitophagy to induce mitochondrial fission in glioma cells. Mitophagy induced by mitochondrial fission can protect glioma cells from apoptosis.^35^ Inhibition of mitophagy and mitochondrial fission sensitizes cancer cells to chemotherapy through DNM1L‐mediated mitochondrial fission.^64^ In the present study, we did not explore the relationship between mitophagy and mitochondrial fission in glioma cells. In the future, we will investigate the mechanism between mitophagy and mitochondrial fission in glioma cells.

Our research found that HAX1 could indeed affect the phosphorylation level of AKT, and increasing the level of phosphorylated AKT can make up for part of the lack of HAX1. At the same time, increasing the level of phosphorylated AKT could promote the phosphorylation of Drp, thereby increasing mitochondrial fission. Therefore, we speculated that HAX1 also has phosphorylation sites, and the phosphorylation of HAX1 affected the phosphorylation of Drp, thereby affecting mitochondrial fission. Phosphorylated AKT play an intermediary role in the middle. However, the in‐depth mechanism of them still needed to be confirmed by another research. Recent studies have reported that HAX1 could protect cancer cell via AKT signal pathway.^22^, ^27^ AKT is widely involved in tumour cell apoptosis. AKT knockdown could reduce the survival of cancer cells by regulating EGFR.^65^ mTOR also acts as a downstream of AKT to regulate cancer cell apoptosis.^66^ In hypoxia, AKT can inhibit cancer cell apoptosis through VEGF.^67^ Furthermore, AKT was reported to repress glioma cell apoptosis through various signal pathway.^68^, ^69^, ^70^, ^71^

Deng X, Guo XB and their colleague have demonstrated that HAX1 regulates glioma tumour through impacting glioma cells and endothelial progenitor cells.^25^, ^26^, ^27^ AKT pathway plays an important role in this process. Our study focuses on the effect of HAX1 on glioma in hypoxic environment. This study provides a new understanding of the mechanism of HAX1 in glioma.

**FIGURE 8:**
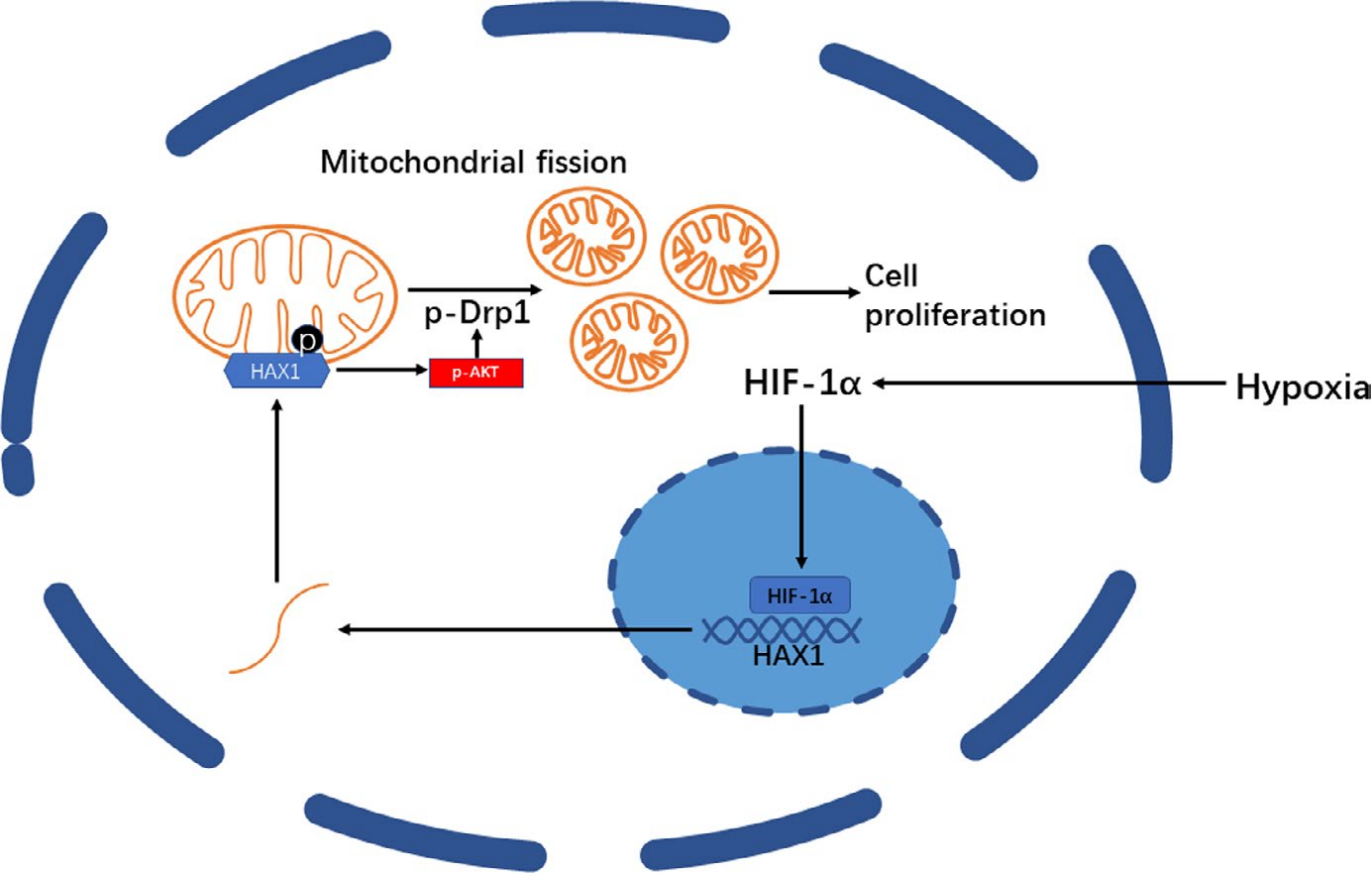
Schematic diagram of the mechanism of HAX1 in glioma. HAX1 promoted glioma cells to adapt to hypoxic environment. HAX1 might increase the phosphorylation of Drp through phosphorylation of AKT to promote mitochondrial fission

In conclusion, our study demonstrated that HAX1 played an important role in glioma cells in hypoxia. HAX1 promoted glioma cells to adapt to hypoxic environment. HAX1 might increase the phosphorylation of Drp through phosphorylation of AKT to promote mitochondrial fission, so as to better adapt to the hypoxic environment (Figure 8). Our research showed how glioma cells adapted to a hypoxic environment, providing potential targets for the treatment of glioma.

## CONFLICT OF INTEREST

All authors declare that there are no conflicts of interest.

## AUTHOR CONTRIBUTION

**Jinghui Lin:** Formal analysis (equal); Investigation (equal); Methodology (equal); Software (equal); Visualization (equal); Writing‐original draft (equal); Writing‐review & editing (equal). **Yang Wang:** Formal analysis (equal); Investigation (equal); Methodology (equal); Software (equal); Writing‐original draft (equal). **Zhiqing Lin:** Conceptualization (lead); Data curation (lead); Funding acquisition (lead); Project administration (lead); Writing‐original draft (equal); Writing‐review & editing (equal).

## Data Availability

The data and materials in this current study are available from the corresponding author on reasonable request.
